# A novel semi‐solidifying liquid formula via the nasogastric route to maintain enteral nutrition in the event of recurrent aspiration pneumonia: A case report

**DOI:** 10.1002/ccr3.1668

**Published:** 2018-07-10

**Authors:** Ezekiel Wong Toh Yoon

**Affiliations:** ^1^ Department of Internal Medicine (Gastroenterology) Hiroshima Kyoritsu Hospital Hiroshima City Japan

**Keywords:** aspiration pneumonia, enteral nutrition, semi‐solid feed, semi‐solidifying liquid formula

## Abstract

Recurrent aspiration pneumonia can impede the continuation of enteral nutrition. Semi‐solid feeds have been demonstrated to reduce the incidence of aspiration pneumonia but are difficult to administer via the nasogastric tube. A novel semi‐solidifying enteral formula may be used instead to avoid the occurrence of severe gastroesophageal reflux.

## 1 INTRODUCTION

Enteral nutrition (EN) is the route of choice for nutrition support when gut integrity is intact, especially in critically ill patients.^1^, ^2^ Feeding‐related adverse events such as aspiration pneumonia, presumably due to severe gastroesophageal reflux, may impede the use of EN. Feeding intolerance, commonly defined as large gastric residual volumes (nasogastric aspirate of >350‐400 mL) along with gastrointestinal symptoms, has been reported to be as high as 40% in severely ill patients.^3^

Semi‐solid feeds have been demonstrated to reduce the risk of aspiration pneumonia and shorten postoperative length of stay after percutaneous endoscopic gastrostomy (PEG).^4^ However, the use of semi‐solid feeds via the nasogastric route is difficult in practice due to the high viscosity and only one case report exists in the literature.^5^ Recently, liquid formulas that increase in viscosity with the help of pectin within the stomach (semi‐solidifying liquid formulas) have been developed. We herein report the successful use of a novel semi‐solidifying liquid formula via the nasogastric route to maintain EN in a patient with recurrent aspiration pneumonia.

## 2 CASE REPORT

**Figure 1:**
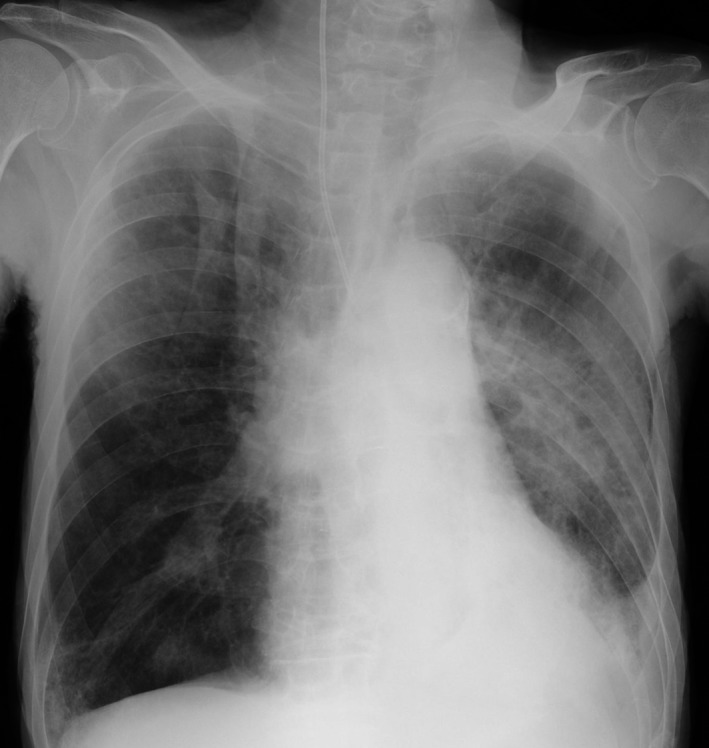
Chest radiograph before transfer to the Department of Internal Medicine

An 84‐year‐old man with a previous history of cerebral infarction, dementia, and symptomatic epilepsy was admitted to our hospital's surgery department due to small bowel obstruction. Decompression via the nasogastric route was successful, but due to poor oral intake and recurrent aspiration pneumonia, he was transferred to our department (Internal Medicine) for further treatment on day 49. During presentation, his body temperature was 39.5°C, heart rate was 120 beats per minute, blood pressure was 101/71 mm Hg, and peripheral oxygen saturation (SpO_2_) was 87% with oxygen administered at 5 L/min by reservoir mask. Coarse crackles were audible on bilateral lung fields (left > right). Laboratory finding revealed leukocytosis (16, 780/μL) with neutrophilia (89%), elevated levels of blood urea nitrogen (64.2 mg/dL), and a high C‐reactive protein level (18.47 mg/dL). Chest radiograph showed pulmonary infiltrates in the left lung (Figure 1).

**Figure 2:**
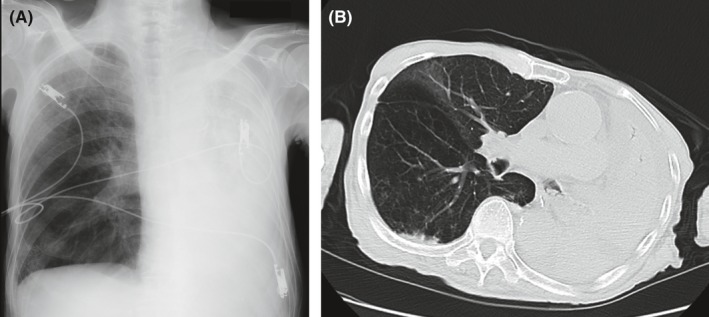
Chest radiograph (A) and CT scan (B) revealing complete atelectasis of the left lung

He was treated with antibiotics (meropenem hydrate 1.5 g/d), which led to some improvement in his fever and SpO_2_. A nasogastric tube was inserted on the 1st day of transfer, and enteral nutrition (EN) with a polymeric liquid formula (1.5 kcal/mL; 40% carbohydrate, 44% lipid, and 16% protein) was initiated on the following day at 40 mL/h (total 375 mL/d). A follow‐up chest radiograph 2 days (3rd day after transfer) after commencing EN did not show any remarkable changes. On the 5th day, however, the patient developed a fever of 38°C and his SpO_2_ decreased to between 70% and 80%. EN was discontinued, and he was placed on parenteral nutrition alone. Chest radiograph (Figure 2A) and CT scan (Figure 2B) revealed complete atelectasis of the left lung with large amount of foreign body (aspirate) blocking the left main bronchus.

**Figure 3:**
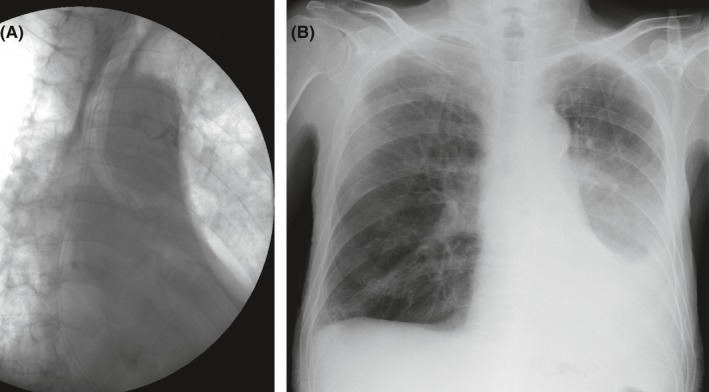
Suction of the left main bronchus using a nasogastric tube (A) and subsequent improvement of atelectasis (B)

Aspiration due to the regurgitation of liquid enteral feed was suspected, but conventional nasal or oral suction was ineffective. On the 7th day, suction was performed by inserting a 15 Fr size nasogastric tube orally into the left main bronchus with the aid of fluoroscopy (Figure 3A). This led to significant improvement in his atelectasis as confirmed by a chest radiograph on the 8th day (Figure 3B). EN was resumed from the 8th day using a novel semi‐solidifying liquid formula (HINE E‐GEL^Ⓡ^, Otsuka Pharmaceutical Factory, Inc., Tokushima, Japan) via the nasogastric route. HINE E‐GEL^Ⓡ^ is a polymeric formula in liquid form with a caloric density of 0.8 kcal/mL (64.2% carbohydrate, 19.8% lipid, and 16% protein) and a dynamic viscosity of about 10 mPa·s (cP), enabling it to be administered easily via a regular nasogastric tube. This formula was administered using gravity control infusion starting at 375 mL/d (300 kcal/d). HINE E‐GEL^Ⓡ^ contains 0.9 g of low‐methoxyl pectin (LMP) per 100 kcal and calcium phosphate. In an acidic environment (gastric lumen), the calcium phosphate ionizes to Ca^2+^ which in turn binds to the LMP. The gelation process causes the dynamic viscosity to increase a hundredfold to more than 1000 mPa·s, giving it a semi‐solid like texture, just like yogurt.

**Figure 4:**
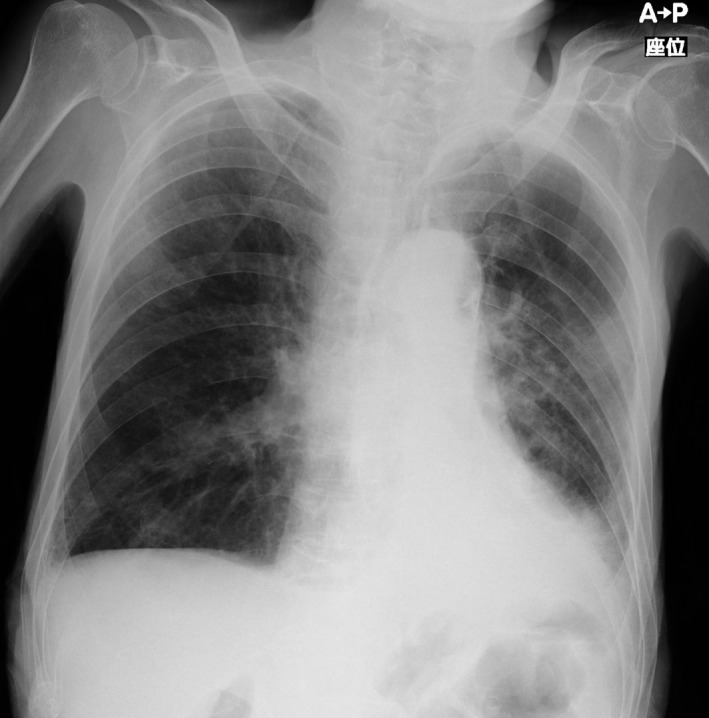
Marked improvement of aspiration pneumonia and atelectasis before discharge

The novel formula was well tolerated, and EN was gradually increased to 900 kcal/d by the 16th day. Unable to achieve adequate oral intake, the patient received PEG tube placement on the 19th day after transfer and was discharged to a long‐term care hospital 3 weeks later without further complications. After PEG, the patient was fed using a regular semi‐solid feed (PG Soft Ace^Ⓡ^, Terumo Corporation, Tokyo, Japan) through his gastrostomy tube. Chest radiograph before discharge showed marked improvement of his pneumonia and atelectasis (Figure 4). Table 1 summarizes the differences (improvement) in various nutritional biomarkers between the 8th day (resumption of EN) and the 33rd day (before discharge).

**Table 1:** Improvement in various nutritional biomarkers after resumption of EN

Nutritional biomarker	8th day (resumption of EN)	33rd day (before discharge)
Serum albumin, g/dL	1.9	2.6
Cholinesterase, U/L	104	148
Total cholesterol, mg/dL	127	155
Total iron binding capacity, μg/dL	95	143
% Total lymphocyte count	11.8	19.0
Onodera's prognostic nutritional index	22	33

## 3 DISCUSSION

In Japan, semi‐solid feeds are often used to deal with feeding‐related adverse events such as aspiration pneumonia, peristomal leakage, and diarrhea in PEG patients. Due to its high viscosity, semi‐solid feed has been reported to reduce the incidence of gastroesophageal reflux and a recent study also showed that it can reduce the risk of aspiration pneumonia in PEG patients.^4^, ^6^

However, as a typical nasogastric tube (8‐15 Fr size for adults) has a lumen that is significantly smaller than a typical PEG tube (15‐24 Fr size for adults), the use of semi‐solid feeds via the nasogastric route is not practical considering that the dynamic viscosity of some semi‐solid feeds may be as high as 20 000 mPa·s. To our knowledge, only one case report exists in the literature where an 18 Fr size nasogastric feeding tube was used to administer the semi‐solid feed.^5^ Although EN was successful and improvement of nutritional status was observed in that case, the use of such large caliber nasogastric feeding tubes is uncommon in Japan due to the extreme discomfort it may cause.

Pectin is a soluble fiber rich in galacturonic acid that gels under specific conditions such as a low‐pH environment (high‐methoxyl pectin or HMP) and the presence of Ca^2+^ ions (LMP).^7^ Adding standard enteral liquid formula to a preadministered LMP solution has been demonstrated to be effective for preventing gastroesophageal reflux events in PEG patients.^8^ Recently, semi‐solidifying liquid formulas are also available in Japan, one of them being HINE E‐GEL^Ⓡ^, which contains LMP and increases in dynamic viscosity when the calcium phosphate in it ionizes to Ca^2+^ in the gastric lumen. When not in contact with Ca^2+^, HINE E‐GEL^Ⓡ^ has a low dynamic viscosity similar to milk and other standard enteral liquid formula, making it easy to administer via a nasogastric tube. The use of this semi‐solidifying formula helped maintain enteral nutrition in our patient until he was stable enough to undergo PEG for long‐term tube feeding.

Jejunal feeding using a feeding tube with gastric decompression function would also be a suitable option to manage this patient after unsuccessful nasogastric feeding.^9^ However, it will require the adjustment or change of feeding tube, not to mention that jejunal placement could be technically challenging even with the aid of fluoroscopy. Contrary to this, the use of semi‐solidifying liquid formulas does not need any adjustment of the nasogastric tube and can be used easily just like other standard liquid formulas. Therefore, patients being fed via the nasogastric route but exhibit symptoms of gastric intolerance with standard liquid formula could benefit from the use of this formula. This would include critically ill patients, patients with a frequent history of aspiration pneumonia and gastroesophageal reflux disease. After establishing a route for long‐term enteral feeding (eg, PEG), patients have the option of using either the same semi‐solidifying formula or a regular semi‐solid feed (as in our case).

Table 2 summarizes the characteristics of different semisolidifying formulas available in Japan, comparing them to a standard liquid feed and a regular semi‐solid feed. Currently, the main semi‐solidifying formulas in Japan are HINE E‐GEL^Ⓡ^ and Mermed Plus^Ⓡ^ (Terumo Corporation). Mermed Plus^Ⓡ^ uses sodium alginate that goes through a gelation process under acidic conditions.

**Table 2:** Characteristics and nutrient data of different semi‐solidifying formulas in comparison with a standard liquid feed and a regular semi‐solid feed

Characteristics (per 100 kcal)	Standard liquid feed^a^	Regular semi‐solid feed^b^	HINE E‐GEL^Ⓡ^	Mermed Plus^Ⓡ^
Total volume, mL	100	126	125	133
Protein, g	4	4	4	4
Fat, g	2.8	2.2	2.2	3.8
Carbohydrate, g	15.5	17.1	16.8	13.6
Dietary fiber, g	1	1.4	1.4	1.1
Water, mL	84.5	110	110	118
Gelling agent	‐	Agar‐agar	Pectin (LMP)	Sodium alginate
Viscosity, mPa·s (or cP)				
In package	5‐10	20 000	10	35
In gastric lumen	5‐10	20 000	>1000^c^	>1000^d^

a Meiji's Mei Balance 1.0 Z^Ⓡ^ (Meiji Holdings Co., Ltd., Tokyo, Japan).

b PG Soft Ace^Ⓡ^ (Terumo Corporation).

c Depending on amount of Ca^2+^ ions present.

d Depending on low‐pH environment (acid secretion in gastric lumen).

## 4 CONCLUSION

In conclusion, we reported the successful use of a novel semi‐solidifying liquid formula via the nasogastric route in a patient with recurrent aspiration pneumonia. This formula helped maintain enteral nutrition in our patient after the use of standard liquid formula was unsuccessful. Semi‐solidifying liquid formulas can be considered as an option for nasogastric feeding in patients with high risk of aspiration pneumonia.

## CONFLICT OF INTEREST

None declared.

## AUTHOR CONTRIBUTIONS

EWTY managed the patient and prepared the manuscript.
